# Soluble expression of proteins correlates with a lack of positively-charged surface

**DOI:** 10.1038/srep03333

**Published:** 2013-11-26

**Authors:** Pedro Chan, Robin A. Curtis, Jim Warwicker

**Affiliations:** 1 Faculty of Life Sciences, 131 Princess Street, Manchester M1 7DN, UK; 2 School of Chemical Engineering and Analytical Sciences, University of Manchester, Manchester Institute of Biotechnology, 131 Princess Street, Manchester M1 7DN, UK; 3 Current address: MyReBase, 5H, On Fook Industrial Building, 41-45 Kwai Fung Crescent, Kwai Chung, New Territories, Hong Kong

## Abstract

Prediction of protein solubility is gaining importance with the growing use of protein molecules as therapeutics, and ongoing requirements for high level expression. We have investigated protein surface features that correlate with insolubility. Non-polar surface patches associate to some degree with insolubility, but this is far exceeded by the association with positively-charged patches. Negatively-charged patches do not separate insoluble/soluble subsets. The separation of soluble and insoluble subsets by positive charge clustering (area under the curve for a ROC plot is 0.85) has a striking parallel with the separation that delineates nucleic acid-binding proteins, although most of the insoluble dataset are not known to bind nucleic acid. Additionally, these basic patches are enriched for arginine, relative to lysine. The results are discussed in the context of expression systems and downstream processing, contributing to a view of protein solubility in which the molecular interactions of charged groups are far from equivalent.

Protein solubility and propensity to aggregate has been central to biotechnology and biosciences through the era of recombinant protein expression. It is also becoming increasingly important in the area of formulation and preparation of biologics (therapeutic proteins), and in consideration of disorders arising from misfolding^1^. A common view of protein aggregation at relatively high concentration holds that partial unfolding (a structural feature) leads to association of non-polar stretches of amino acids (a sequence feature). Whilst structural and sequence properties combine in this view of protein aggregation, computational algorithms that attempt to predict solubility largely divide into those based on sequence and those based on structure, although some features (e.g. net charge) span this division.

A key question relates to how solubility is defined in benchmark sets. Early work^2^ distinguishes between proteins that form inclusion bodies (IBs) and those that do not, with a study of sequence features. The two properties correlating best with IB formation were found to be average charge (more net charge, less IB), and turn-forming residue fraction (more gives IBs, perhaps due to slow folding e.g. with prolines). Other work^3^ also uses the IB/non-IB distinction, together with sequence and structure-based correlations with solubility, including thermostability and relative lack of β-sheet. Some reports define soluble proteins as those for which a structure has been solved and deposited in the protein data bank, PDB^4^. This definition is used alongside resources that record progress in protein expression for structural genomics^5^,^6^, such as the TargetDB database^7^. Machine-learning techniques are then employed to optimise distinction between soluble and insoluble proteins, although it can be difficult to extract physico-chemical interpretation from such methods. Other work combines machine-learning with soluble/insoluble datasets obtained through keyword searching in the literature^8^. The relationship between mRNA levels and protein solubility in *E. coli* has been examined^9^. Proteins with sequence more prone to aggregation are generally expressed at lower levels, where amino acid polarity is used to indicate aggregation potential, i.e. proteins with a more non-polar sequence have lower mRNA levels. The REFOLD database^10^ annotates proteins as soluble or insoluble, but in practice all of these proteins have been expressed through IB formation.

There are a number of aggregation prediction schemes based on the experimental observation that many proteins can be induced to adopt an amyloid, β-rich conformation^11^. These include TANGO^12^, PASTA^13^, and Zyggregator^14^. Such schemes can include many factors, but generally, the β-forming propensity for linear segments of amino acid sequence is an important element. A 3D surface polarity approach has been adopted in the redesigning of protein surface to improve solubility^15^, with the introduction of groups to break up non-polar patches. This is reminiscent of the discovery that charges on the surfaces of hyperthermophile proteins are more closely packed, on average, than those in mesophile proteins^16^. It was assumed that the higher temperature environment of hyperthermophiles increases the strength of hydrophobic interactions, leading to the requirement for a more stringent breaking up of non-polar patches with charges. A charge influence has also appeared in the context of translation rate, a property that will impact on protein production and therefore potentially solubility. A dependence of ribosomal velocity on positively charged residues in newly synthesised proteins has been found, due to interaction with the negatively-charged ribosomal exit tunnel^17^. More generally, translation rate has a well-studied correlation with codon bias^18^.

Methods for predicting protein solubility have been reviewed^19^. The availability of experimental data, where proteins have been expressed in consistent conditions, continues to present a significant problem with assessing prediction schemes. A significant study addressing this point used a high throughput cell-free system for classification of *E. coli* protein solubility^20^. The authors of this work concluded that factors correlating to some degree with solubility include charge and structural class, whilst algorithms based largely on propensity to form β-structure/amyloid performed less well, although a machine-learning study subsequently identified a correlation between sequence-based calculation of physico-chemical properties and measured solubility for this dataset^21^.

In the current work, computational methods for characterising charge and potential distributions in proteins^22^ have been used alongside patch-based calculations of surface properties^23^ to analyse the properties of soluble and insoluble subsets of proteins. The experimental data used in this study derive from cell-free expression^20^ using the PURE system of *E. coli* factors, lacking chaperones^24^. Encouraged by a study in which computation over many proteins revealed a correlation between electrostatic properties and subcellular location^25^, a similar approach was used in respect of solubility. Whilst some correlation is found between insolubility and larger non-polar patches, by far the most significant relationship associates insolubility with large positively-charged patches. The pattern underlying this unexpected result is similar to that which separates nucleic acid (NA)-binding from non-NA-binding proteins.

## Results

### Surface potential patches and solubility

**Figure 1:**
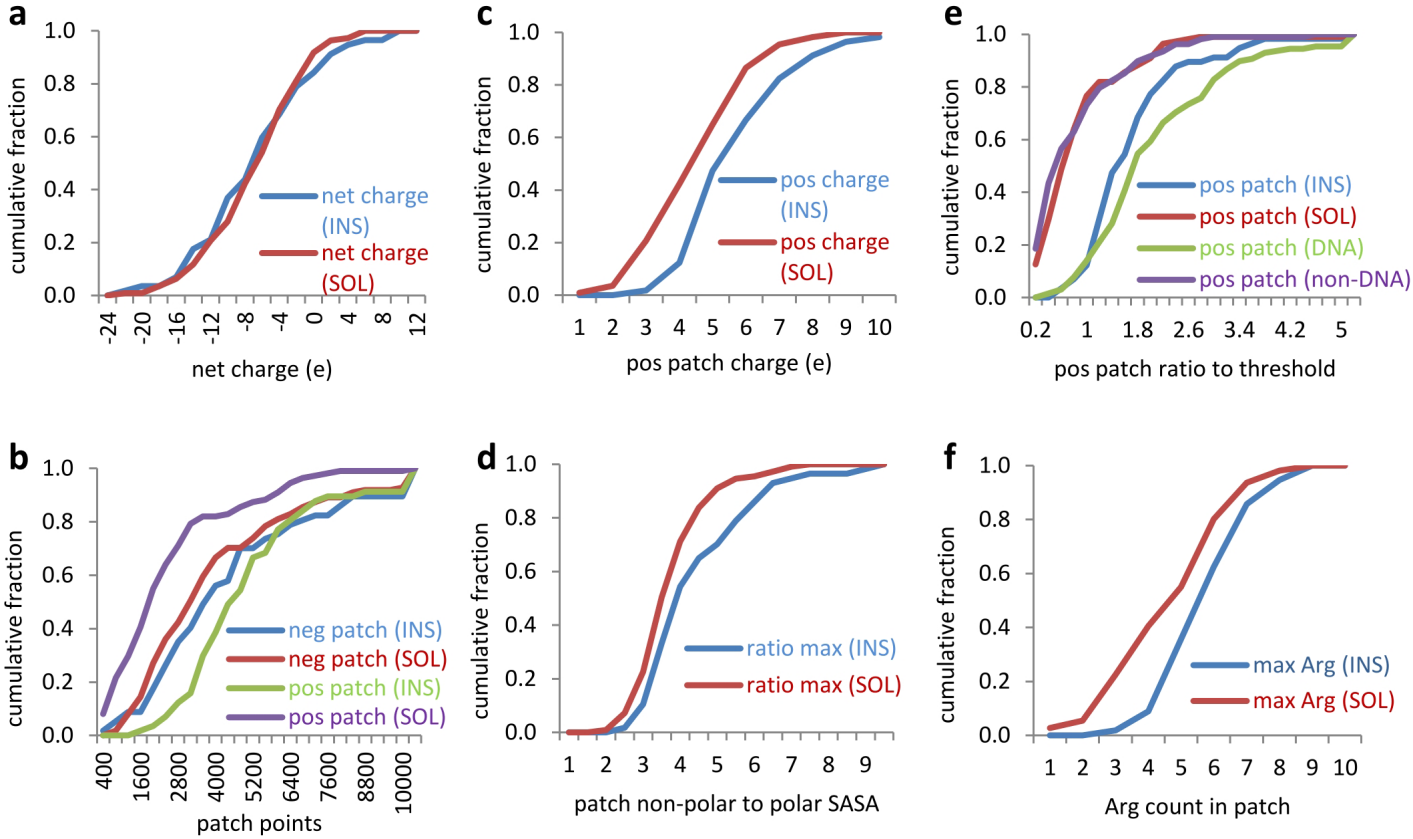
Cumulative fractions of soluble (SOL) and insoluble (INS) protein datasets, upon calculation of particular features. (a) Net charge, predicted at pH 7.0. (b) Grid points within the largest positive (pos) and largest negative (neg) contours of electrostatic potential. (c) Maximum net positive charge in a geometric patch (13 Å radius). (d) The maximum ratio (for each protein) of non-polar to polar patch SASA. (e) Largest positive patch contours are re-plotted, now as a ratio to a 3000 grid point threshold, alongside calculations with DNA-binding and non-DNA-binding datasets. (f) Separation according to the geometrical patch with the largest Arg content.

At neutral pH most of the insoluble and soluble dataset proteins are predicted to be moderately negatively-charged, and there is no significant separation of the distributions (Fig. 1a, p = 0.872 for a Mann-Whitney test of subsets being sampled from the same underlying distribution). The maximal positive and negative potential patches for each protein show quite different behaviour, with no significant separation for negative potential (p = 0.227), but clear separation for positive potential (p = 7.1 × 10^−13^, Fig. 1b). A patch analysis of charge clustering (with 13 Å patch radius) was performed in order to establish whether the positive potential patches, based on contours, were mirrored in charge geometry. This is the case, with the largest net positive charge on a patch also distinguishing soluble and insoluble protein datasets (p = 2.2 × 10^−4^, Fig. 1c).

### Surface polarity and solubility

**Figure 2:**
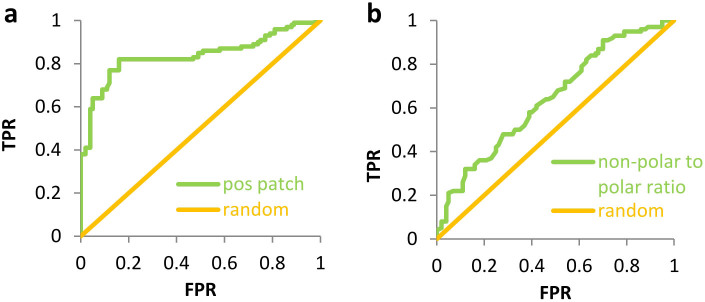
ROC plots for insoluble and soluble subset separation. (a) ROC plot (AUC = 0.85) showing separation by positive potential. TPR is true positive rate and FPR false positive rate. (b) ROC plot (AUC = 0.62) quantifying the separation by non-polar to polar surface ratio (13 Å radius patch).

We next examined a potential role for the association of proteins via non-polar surfaces, through calculation of non-polar to polar solvent accessible surface area (SASA) ratios, for patches of radius 13 Å centred on each atom. The maximum of this ratio was identified for each protein. Fig. 1d shows the separation of soluble and insoluble subsets (p = 2.3 × 10^−3^). Whilst there is some correlation between increased non-polarity and insolubility, it is far smaller than that exhibited by positive potential. ROC plot analysis demonstrates this distinction (Fig. 2). An area under the curve (AUC) of 0.85 for the positive potential features (Fig. 2a) compares with an AUC of 0.62 for the non-polar to polar surface area ratio (Fig. 2b). A threshold of 3000 grid points (in the contours of positive potential) gives the best separation between soluble and insoluble datasets. Positive patch size can be reported as a ratio to this value.

### Comparison with discrimination for DNA-binding proteins

Positively-charged surfaces are implicated in nucleic acid binding^26^, and contribute to prediction schemes for NA binding^27^. The same potential patch analysis applied to the solubility data, was used to examine DNA-binding and non-DNA-binding protein datasets. Separation of the DNA/non-DNA-binding subsets is strikingly similar to that for the insoluble/soluble subsets (Fig. 1e). There is some enrichment for known DNA-binding proteins in the insoluble subset, 13 of 56, as compared with 16 of 111 in the soluble subset. Clearly though not all DNA-binding proteins are present in the insoluble subset.

Generally in NA binding, non-specific charge interactions typically function alongside more specific interactions arising from hydrogen-bonding and shape complementarity. Such additional interactions may play a role in distinguishing an interesting pair of DNA-binding homologues, IHF in the insoluble subset and HUα in the soluble subset. Maximal positive patches (measured as a ratio to the threshold) are consistent with the subset membership, whether calculated for the monomer (IHF 1.38, HUα 0.26) or the dimeric biological units (IHF 3.31, HUα 0.45). Although these two proteins are closely related structurally, they have very different positive potential distributions and solubility in the cell-free system. Functionally, HUα and IHF have divergent DNA substrate preferences^28^, that may be related to their positive charge distributions.

It appears that larger positive patches exert an influence towards protein aggregation in the cell free expression system^20^. If this is also the case for intracellular expression, then it might be anticipated that it would be countered by a cell maintaining lower levels of proteins with larger positive potential patches. Abundance of mRNA is not entirely representative of protein level^29^. Indeed, at a fixed time point for a single cell, correlation between protein and mRNA level can be absent, due to the much shorter lifetime of mRNAs as compared with proteins^30^. Currently though, mRNA levels measured from populations of cells, which are correlated with protein level^30^, provide the most extensive data. An anti-correlation (R = 0.283, p = 5.75 × 10^−3^, not shown) was found between largest positive patch size and a log measure of mRNA levels in *E. coli*^31^. Again the positive potential is differentiated from negative potential, for which the largest patch gives no significant relationship with mRNA level (R = 0.124, p = 0.138, not shown).

### Positive and negative charge, arginine and lysine

Sequence-based calculation of the fraction of charged groups that are either positively-charged or negatively-charged at neutral pH separates soluble and insoluble subsets (p = 1.417 × 10^−3^, not shown). Consistent with the patch calculations, a higher fraction of positive charge tends towards insolubility. With a greater separation of soluble and insoluble subsets for the (3D) patch-based property, relative to the sequence-based charge fraction, the structural property appears to be a crucial component in a physico-chemical understanding of the cell-free expression data.

Thus far, positively-charged amino acid sidechains, at neutral pH, have been combined. Taking the maximum positive charge patches of Fig. 1c, calculation of Arg enrichment in these patches (compared with the Arg to Lys content overall for each protein) gives a separation (p = 0.023, not shown), with more Arg in the maximum positive patches of the insoluble subset. Next, looking at the maximum number of Arg in any geometric patch of radius 13 Å, there is also separation (p = 3.884 × 10^−4^, Fig. 1f). Only a small number of the insoluble proteins have a geometric patch containing less than 4 Arg. The Lys to Arg ratio calculated directly from protein sequence also separates (p = 0.037, not shown), with very few of the insoluble dataset having this ratio greater than 1.

## Discussion

Our results indicate that factors contributing on average to separation of the structurally annotated soluble and insoluble subsets in cell-free expression^20^, are non-polar surface (moderate contribution), and positively-charged patches (major contribution, particularly where Arg is more prevalent than Lys). Correlation between largest positive patch and insolubility implies that this property, or another feature to which it is strongly related, acts in some direct or indirect way to promote protein-protein interactions. It could be argued that a concentration of positive charge may tend towards lower folded state stability through unfavourable charge interactions, and thus influence solubility via (partial) unfolding. However, a similar influence would be expected for negatively-charged patches, which is absent. Through what other mechanisms could positive charge clustering contribute to insolubility? Given that the characteristic for insolubility observed in the current work closely matches that for NA-binding proteins, a hypothetical mechanism based on an intermediate step of binding to NA is presented. This model applies only to media rich in NA, such as during expression. A second area of discussion is around the growing literature on negative charge and the solubility of purified proteins.

**Figure 3:**
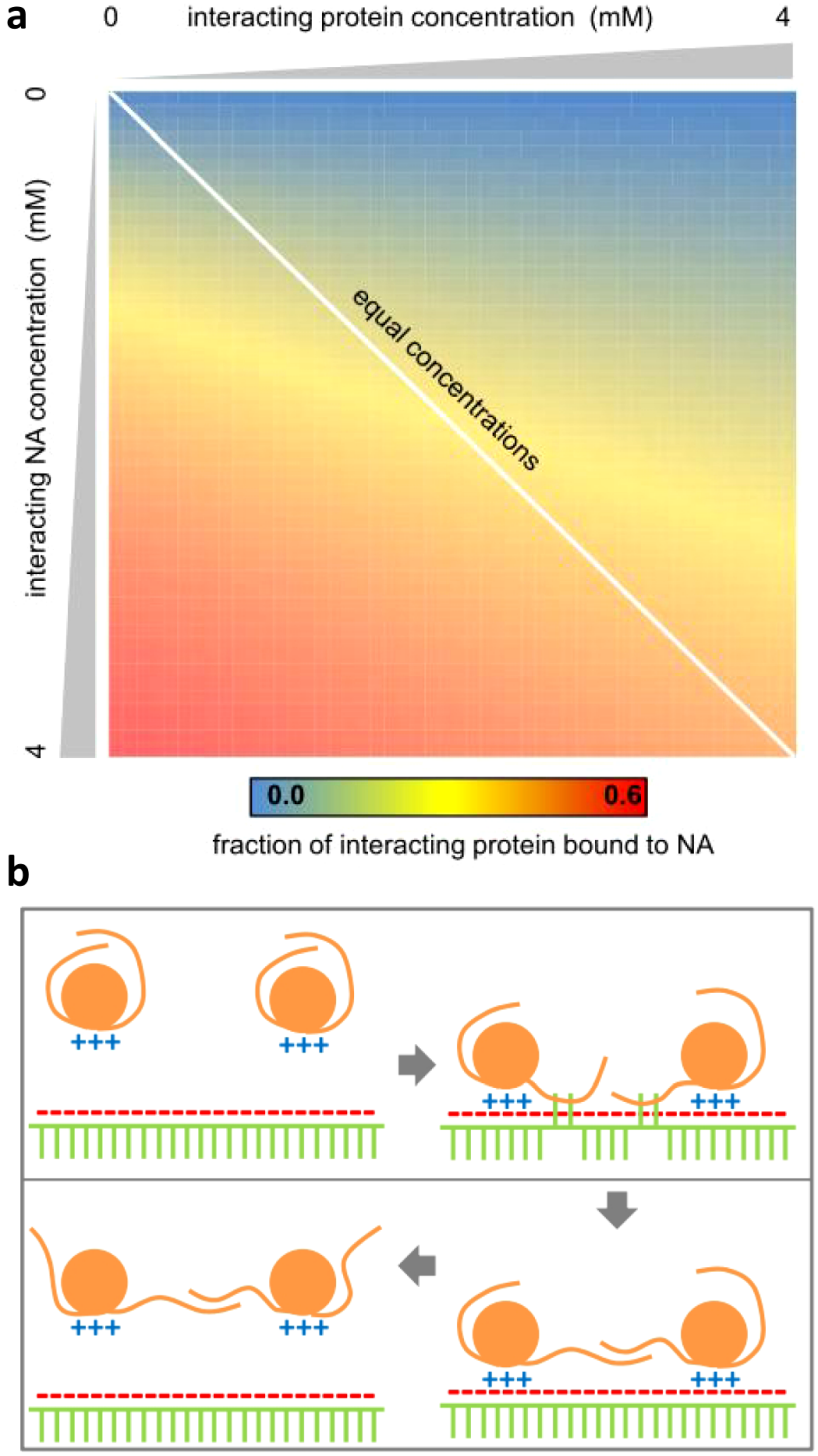
Weak interactions and association in a crowded environment. (a) Two species interact with an energy of 15 kJ/mole. Concentrations are varied (0 to 4 mM) for protein interacting sites (horizontally) and NA interacting sites (vertically). The heat map shows the proportion of interacting protein sites that are complexed (scale bar under the map). See text for more detail. (b) A hypothetical scheme is drawn in which protein-NA interactions are mediated by charge interactions (upper left), followed by partial unfolding concomitant with NA base – protein interactions (upper right), then protein-protein association through non-polar interactions (lower right), and finally dissociation of protein from NA (lower left).

Fig. 3a shows an equilibrium model for protein binding to nucleic acid, based on a relatively weak interaction of 15 kJ/mole. This corresponds to 3 close salt-bridges or greater than 3 flexible +/− charge interactions, consistent with a net charge threshold of about 4.5 (Fig. 1c). Briefly, having estimated maximal concentrations of charged protein (positive) and NA (negative) sites each at 4 mM, concentration ramps up to the these values are used to account for subsets of protein and NA sites possessing the appropriate unshielded charge (see Methods for more detail). This simple calculation (Fig. 3a) shows that a weak interaction, coupled with relatively high concentrations, leads to a substantial fraction of (transient) complexes. The diagonal drawn on Fig. 3a relates to equal concentrations of interacting components, and thus also applies to the case of direct protein-protein interactions.

Fig. 3a does not address the issue of how charge-based complexation between protein and nucleic acid might lead to protein insolubility. Available data indicate that nucleic acid constitutes at most a small fraction of inclusion body material for proteins expressed in *E. coli*^32^, although nucleic acid can affect the rate of aggregation^33^. In Fig. 3b, a scheme is outlined that indicates how transient protein-nucleic acid interactions could lead to a lowering of the activation energy for folding/unfolding transitions, thereby accelerating protein-protein complexation and insolubility if this complexed state ultimately leads to kinetically trapped aggregates. Increased polyanion hydrophobicity leads to a reduction in protein stability in protein-polyanion complexes^34^. Nucleic acids have a substantial non-polar component and Fig. 3b schematises non-polar interactions between bases and partially unfolded regions of protein. This could lead to protein-protein interactions if partially unfolded proteins transiently bound to the polyanion are adjacent to each other. Such a mechanism could contribute to seeding protein aggregation, effectively concentrating a population of proteins undergoing folding transitions, through transient condensation onto polyanions.

A study of net charge, within sequence windows of 21 amino acids in the yeast proteome, found that larger net positive charge was substantially under-represented in comparison with the equivalent net negative charge^35^, and when present was often associated with NA binding. This is consistent with net positive charge on proteins being moderated unless functionally associated with nucleic acid binding, perhaps to avoid pathways such as that hypothesised in Fig. 3b. Generally, NA-binding proteins such as transcription factors can be difficult to express^36^,^37^.

It is worth stating the fundamental points that relate protein surface charges to protein solubility. The hydration of charged groups is correlated with protein solubility in aqueous solutions^38^. Beyond this, solubility often decreases near to the isoelectric point as net charge and electrostatic repulsion decreases, allowing non-specific attractive interactions to form. Additionally, near to the pI, proteins with anisotropic charge distributions sample attractive interactions between patches of opposite charge, which are screened with increasing ionic strength^39^. Within this general framework, there are several reports that bear on the relative role of positive and negative surface charges in protein solubility. A strong preference for Asp/Glu over Lys/Arg was observed in a phage display screen for substitutions that enhance resistance to aggregation in human antibody variable domains^40^. Addition of an acidic tag to a positively-charged intrabody enhances expression^41^. It has been reported that many chaperones possess regions of negative charge, and that acidic regions modulate the anti-aggregation activity of Hsp90^42^. The current work suggests that surface charge chaperoning may be a contributing factor. A growing body of data is becoming available with which to test such hypotheses^43^.

Solubility measurements for 7 proteins in different precipitants show that negative surface charge correlates with increased solubility, independent of the nature of the precipitant^44^. These experiments, which reflect protein-protein interactions between folded (purified) proteins, are quite different to the cell-free translation study^20^ on which the current work is based, but given the importance of charge, we made patch calculations for these 7 proteins. No correlation is seen for maximum positive patch size and solubility (R = 0.393, p = 0.191, not shown), but a relationship may be present between overall Lys to Arg ratio and solubility (R = 0.720, p = 0.034, not shown), with (again) a higher ratio tending towards more soluble. One interpretation is that the Arg sidechain is particularly prone to interactions. Solubility in the cell-free system could be related more to the avoidance of basic clusters (and perhaps NA binding), whereas the purified protein experiments may be probing, in part, a more general stickiness associated with the Arg sidechain. The authors of the precipitant-based solubility study concluded that strong water binding by acidic amino acids may underpin the results^44^. The balance between negative and positive amino acid sidechain charges in fine-tuning solubility, remains to be established.

It is of interest that Arg has been identified as a ubiquitous interacting amino acid in informatics studies, with an elevated propensity (relative to average surface occurrence) for interfaces in both protein-protein and protein-NA complexes^45^,^46^. Cation – π interactions, involving Arg, are common at protein-protein interfaces^47^, and Arg is also common in protein crystal contacts at low ionic strength^48^. Arginine content of antigen-combining sites in antibodies is correlated with increased non-specific binding^49^. The excipient properties of Arg are also of interest. Solutions of Arg can be effective in solubilising proteins^50^, an effect that becomes more pronounced in mixtures with Glu^51^. This solubility enhancement is related to an increase in the number of Arg and Glu molecules forming interactions with the protein^52^. The interacting properties of Arg cover a range of systems. We suggest that such diversity may lead to a correlation of Arg enrichment with insolubility, whether clustering into patches (with Lys) for polyanion binding, or more generally over a protein surface.

Reduction of positive patches should be of use as a design tool for expression systems, and substituting Arg with other charges could aid the maintenance of high concentrations of purified protein in solution. The hypothesis of protein basic patch interactions with NA in expression systems could be investigated with uncoupling of transcription and translation, to vary relative mRNA and protein levels^53^. There is much yet to establish about the association between basic clusters, and Arg enrichment, and insolubility, given for example the report that green fluorescent protein engineered to bear high net positive or negative charge expresses in *E. coli* and is much more soluble than wild-type protein^54^. In this case perhaps the extreme net charges provide sufficient repulsive interactions to overcome other effects.

## Methods

### Soluble and insoluble datasets and DNA-binding/non-binding protein datasets

Subsets for soluble and insoluble *E. coli* protein expression in the cell-free system were defined following the authors' description^20^. Specifically, soluble proteins are those with a solubility of more than 70%, and insoluble with a solubility of less than 30%. Percentage solubilities had been obtained, following cell-free expression of radiolabelled protein, as the ratio of soluble protein (supernatant from a centrifugation step) and total protein^20^. Members of the soluble and insoluble subsets with structures in the PDB^4^ were obtained through cross-referencing with UniProt^55^. A further filtering step was applied with a cull for sequence identity at a 90% identity threshold, using the PISCES tool^56^. This procedure allows the retention of homologues, since they may have different surface charge and polarity distributions. Final subsets of 111 (soluble) and 56 (insoluble) *E. coli* proteins were available for processing.

Sets of DNA-binding and non-binding proteins were obtained from earlier work^57^,^58^. Most of these PDB ids were accessible and ran successfully through the electrostatic potential patch analysis (128 DNA-binding proteins, 108 non-binders). Calculations were also made for a set of 7 proteins for which solubility data were available in precipitant studies^44^, using the same PDB ids specified in that work.

### Charge, potential and polarity calculations

For polarity analysis, a sphere of radius 13 Å was centred on each non-hydrogen atom. Polar and non-polar solvent accessible surface area was then summed for all non-hydrogen atoms within that sphere, using a 1.4 Å radius solvent probe and polar/non-polar character assigned according to atom type and functional group^23^. The relative polarity of a patch is then calculated as the ratio of non-polar SASA to polar SASA, and the maximum value of this ratio (i.e. most non-polar region) recorded for each protein. When the polar and non-polar SASAs are summed for each patch, the average of this distribution over patches is about 1300 Å^2^. In comparison, a typical evolved interface between proteins buries about 1600 Å^2^ in total, although this is quite variable^59^. Considering that the entirety of each of the two contributing surfaces will not be buried in an association, a patch radius of 13 Å seems reasonable in generating a footprint for non-specific protein-protein interactions.

Electrostatic potential was calculated around each protein using a Finite Difference Poisson-Boltzmann methodology^22^, with negatively-charged Asp, Glu sidechains and C-termini, and positively-charged Lys, Arg and N-termini. Ionic strength was 0.15 M in the calculations. The resulting potential map was contoured at thresholds of +/− kT/e. Importantly, the contours were drawn on a single shell of the calculation grid (on the solvent side of the protein), so that the number grid points in each contoured patch effectively represents the size of that patch. Grid step for electrostatic potential calculation was a constant 0.6 Å, independent of protein. A parallel approach was introduced to confirm that positive charge location underpins the contours of positive potential. The patch analysis described for surface polarity was also used to record the maximum net charge within a geometrical patch. For this purpose, sidechain charges were approximated at Cβ atoms to minimise the effects of sidechain conformational variation.

Receiver Operator Characteristic (ROC) plots were generated for the ability of calculated features to discriminate between soluble and insoluble proteins. As the numerical value of a feature is varied and applied as a threshold to the datasets, corresponding true positive rates (TPRs) and false positive rates (FPRs) are calculated and given in a ROC plot. Area Under the Curve (AUC) is used to estimate effectiveness for separating datasets, with 1.0 equating to complete separation and 0.5 to random. The Mann-Whitney U test was applied to the calculated feature subsets. The probability of occurrence of these particular feature values, if there is no difference in the underlying distributions, is given. A significant difference is inferred if this probability is < 0.05.

To investigate whether a relationship exists between calculated features and expression at the mRNA level, protein IDs for soluble and insoluble datasets were mapped to mRNA abundances for *E. coli* proteins^31^.

### A model for non-specific protein-nucleic acid charge interactions

The model of Fig. 3a is based on a 15 kJ/mole interaction, or about 3 salt-bridges, since typical pKa shifts for a surface salt-bridge are 1 pH unit or 5–6 kJ/mole^60^. Total concentration of protein was calculated for an estimate of 2.35 × 10^6^ protein molecules^61^ in an *E. coli* cell of side 1 μm, giving 4 mM. Summing the estimated contributions of tRNAs and mRNAs^61^ and comparing with protein molecular weight, gives a ratio of about 1:10, nucleic acid to protein. Each protein molecule, of average molecular weight 40 kD^61^, might bind to a polyanion through a single positive patch, whereas each polyanion nucleic acid molecule has multiple binding sites. With a single base molecular weight of about 0.32 kD, assuming that binding sites could recur approximately every 12 bases, then two factors of 10 approximately cancel and the maximum concentrations of positively and negatively-charged binding sites are roughly equal. Although 4 mM is set as this maximum, only a subset of proteins will exhibit positive patches above a certain threshold, whilst there are many factors that will contribute to structuring of nucleic acids and the masking of negative charge. Thus linear concentration ramps, up to 4 mM, are applied in Fig. 3a for interacting subsets of protein and nucleic acid. The heat map is then generated as the proportion of the interacting subset of proteins that is bound to nucleic acid, as the concentrations are altered, given the 15 kJ/mole interaction.

## References

[b1] Vendruscolo M., Knowles T. P. & Dobson C. M. Protein solubility and protein homeostasis: a generic view of protein misfolding disorders. Cold Spring Harb Perspect Biol 3, a010454 (2011). 21825020 10.1101/cshperspect.a010454 PMC3225949

[b2] Wilkinson D. L. & Harrison R. G. Predicting the solubility of recombinant proteins in Escherichia coli. Biotechnology (N Y) 9, 443–448 (1991). 1367308 10.1038/nbt0591-443

[b3] Idicula-Thomas S. & Balaji P. V. Correlation between the structural stability and aggregation propensity of proteins. In Silico Biol 7, 225–237 (2007). 17688448

[b4] Berman H. M. *et al.* The Protein Data Bank. Nucleic Acids Res 28, 235–242 (2000). 10592235 10.1093/nar/28.1.235 PMC102472

[b5] Smialowski P. *et al.* Protein solubility: sequence based prediction and experimental verification. Bioinformatics 23, 2536–2542 (2007). 17150993 10.1093/bioinformatics/btl623

[b6] Magnan C. N., Randall A. & Baldi P. SOLpro: accurate sequence-based prediction of protein solubility. Bioinformatics 25, 2200–2207 (2009). 19549632 10.1093/bioinformatics/btp386

[b7] Chen L., Oughtred R., Berman H. M. & Westbrook J. TargetDB: a target registration database for structural genomics projects. Bioinformatics 20, 2860–2862 (2004). 15130928 10.1093/bioinformatics/bth300

[b8] Niu X., Li N., Chen D. & Wang Z. Interconnection between the protein solubility and amino acid and dipeptide compositions. Protein Pept Lett 20, 88–95 (2013). 22789104

[b9] Tartaglia G. G., Pechmann S., Dobson C. M. & Vendruscolo M. A relationship between mRNA expression levels and protein solubility in E. coli. J Mol Biol 388, 381–389 (2009). 19281824 10.1016/j.jmb.2009.03.002

[b10] Chow M. K. *et al.* REFOLD: an analytical database of protein refolding methods. Protein Expr Purif 46, 166–171 (2006). 16150607 10.1016/j.pep.2005.07.022

[b11] Chiti F. & Dobson C. M. Amyloid formation by globular proteins under native conditions. Nat Chem Biol 5, 15–22 (2009). 19088715 10.1038/nchembio.131

[b12] Linding R., Schymkowitz J., Rousseau F., Diella F. & Serrano L. A comparative study of the relationship between protein structure and beta-aggregation in globular and intrinsically disordered proteins. J Mol Biol 342, 345–353 (2004). 15313629 10.1016/j.jmb.2004.06.088

[b13] Trovato A., Seno F. & Tosatto S. C. The PASTA server for protein aggregation prediction. Protein Eng Des Sel 20, 521–523 (2007). 17720750 10.1093/protein/gzm042

[b14] Tartaglia G. G. & Vendruscolo M. The Zyggregator method for predicting protein aggregation propensities. Chem Soc Rev 37, 1395–1401 (2008). 18568165 10.1039/b706784b

[b15] Chennamsetty N., Voynov V., Kayser V., Helk B. & Trout B. L. Design of therapeutic proteins with enhanced stability. Proc Natl Acad Sci U S A 106, 11937–11942 (2009). 19571001 10.1073/pnas.0904191106 PMC2715526

[b16] Greaves R. B. & Warwicker J. Mechanisms for stabilisation and the maintenance of solubility in proteins from thermophiles. BMC Struct Biol 7, 18 (2007). 17394655 10.1186/1472-6807-7-18 PMC1851960

[b17] Charneski C. A. & Hurst L. D. Positively charged residues are the major determinants of ribosomal velocity. PLoS Biol 11, e1001508 (2013). 23554576 10.1371/journal.pbio.1001508 PMC3595205

[b18] Goetz R. M. & Fuglsang A. Correlation of codon bias measures with mRNA levels: analysis of transcriptome data from Escherichia coli. Biochem Biophys Res Commun 327, 4–7 (2005). 15629421 10.1016/j.bbrc.2004.11.134

[b19] Weiss W. F. t., Young T. M. & Roberts C. J. Principles, approaches, and challenges for predicting protein aggregation rates and shelf life. J Pharm Sci 98, 1246–1277 (2009). 18683878 10.1002/jps.21521

[b20] Niwa T. *et al.* Bimodal protein solubility distribution revealed by an aggregation analysis of the entire ensemble of Escherichia coli proteins. Proc Natl Acad Sci U S A 106, 4201–4206 (2009). 19251648 10.1073/pnas.0811922106 PMC2657415

[b21] Agostini F., Vendruscolo M. & Tartaglia G. G. Sequence-based prediction of protein solubility. J Mol Biol 421, 237–241 (2012). 22172487 10.1016/j.jmb.2011.12.005

[b22] Warwicker J. Continuum dielectric modelling of the protein-solvent system, and calculation of the long-range electrostatic field of the enzyme phosphoglycerate mutase. J Theor Biol 121, 199–210 (1986). 2432357 10.1016/s0022-5193(86)80093-5

[b23] Cole C. & Warwicker J. Side-chain conformational entropy at protein-protein interfaces. Protein Sci 11, 2860–2870 (2002). 12441384 10.1110/ps.0222702 PMC2373749

[b24] Shimizu Y. *et al.* Cell-free translation reconstituted with purified components. Nat Biotechnol 19, 751–755 (2001). 11479568 10.1038/90802

[b25] Chan P. & Warwicker J. Evidence for the adaptation of protein pH-dependence to subcellular pH. BMC Biol 7, 69 (2009). 19849832 10.1186/1741-7007-7-69 PMC2770037

[b26] Warwicker J., Engelman B. P. & Steitz T. A. Electrostatic calculations and model-building suggest that DNA bound to CAP is sharply bent. Proteins 2, 283–289 (1987). 2834718 10.1002/prot.340020404

[b27] Chen Y. C., Wright J. D. & Lim C. DR_bind: a web server for predicting DNA-binding residues from the protein structure based on electrostatics, evolution and geometry. Nucleic Acids Res 40, W249–256 (2012). 22661576 10.1093/nar/gks481 PMC3394278

[b28] Swinger K. K. & Rice P. A. IHF and HU: flexible architects of bent DNA. Curr Opin Struct Biol 14, 28–35 (2004). 15102446 10.1016/j.sbi.2003.12.003

[b29] de Sousa Abreu R., Penalva L. O., Marcotte E. M. & Vogel C. Global signatures of protein and mRNA expression levels. Mol Biosyst 5, 1512–1526 (2009). 20023718 10.1039/b908315d PMC4089977

[b30] Taniguchi Y. *et al.* Quantifying E. coli proteome and transcriptome with single-molecule sensitivity in single cells. Science 329, 533–538 (2010). 20671182 10.1126/science.1188308 PMC2922915

[b31] Bernstein J. A., Khodursky A. B., Lin P. H., Lin-Chao S. & Cohen S. N. Global analysis of mRNA decay and abundance in Escherichia coli at single-gene resolution using two-color fluorescent DNA microarrays. Proc Natl Acad Sci U S A 99, 9697–9702 (2002). 12119387 10.1073/pnas.112318199 PMC124983

[b32] Valax P. & Georgiou G. Molecular characterization of beta-lactamase inclusion bodies produced in Escherichia coli. 1. Composition. Biotechnol Prog 9, 539–547 (1993). 7764166 10.1021/bp00023a014

[b33] Maachupalli-Reddy J., Kelley B. D. & De Bernardez Clark E. Effect of inclusion body contaminants on the oxidative renaturation of hen egg white lysozyme. Biotechnol Prog 13, 144–150 (1997). 9104038 10.1021/bp970008l

[b34] Sedlak E., Fedunova D., Vesela V., Sedlakova D. & Antalik M. Polyanion hydrophobicity and protein basicity affect protein stability in protein-polyanion complexes. Biomacromolecules 10, 2533–2538 (2009). 19645440 10.1021/bm900480t

[b35] Cawley A. & Warwicker J. eIF4E-binding protein regulation of mRNAs with differential 5'-UTR secondary structure: a polyelectrostatic model for a component of protein-mRNA interactions. Nucleic Acids Res 40, 7666–7675 (2012). 22718971 10.1093/nar/gks511 PMC3439904

[b36] Mossakowska D. E. Expression of nuclear hormone receptors in Escherichia coli. Curr Opin Biotechnol 9, 502–505 (1998). 9821279 10.1016/s0958-1669(98)80036-0

[b37] Yang W. C., Welsh J. P., Lee J., Cooke J. P. & Swartz J. R. Solubility partner IF2 Domain I enables high yield synthesis of transducible transcription factors in Escherichia coli. Protein Expr Purif 80, 145–151 (2011). 21757009 10.1016/j.pep.2011.06.017 PMC3183262

[b38] Dumetz A. C., Chockla A. M., Kaler E. W. & Lenhoff A. M. Effects of pH on protein-protein interactions and implications for protein phase behavior. Biochim Biophys Acta 1784, 600–610 (2008). 18258214 10.1016/j.bbapap.2007.12.016

[b39] Neal B. L., Asthagiri D., Velev O. D., Lenhoff A. M. & Kaler E. W. Why is the osmotic second virial coefficient related to protein crystallization? Journal of Crystal Growth 196, 377–387 (1999).

[b40] Dudgeon K. *et al.* General strategy for the generation of human antibody variable domains with increased aggregation resistance. Proc Natl Acad Sci U S A 109, 10879–10884 (2012). 22745168 10.1073/pnas.1202866109 PMC3390889

[b41] Kvam E., Sierks M. R., Shoemaker C. B. & Messer A. Physico-chemical determinants of soluble intrabody expression in mammalian cell cytoplasm. Protein Eng Des Sel 23, 489–498 (2010). 20378699 10.1093/protein/gzq022 PMC2865363

[b42] Wayne N. & Bolon D. N. Charge-rich regions modulate the anti-aggregation activity of Hsp90. J Mol Biol 401, 931–939 (2010). 20615417 10.1016/j.jmb.2010.06.066 PMC2929759

[b43] Ahn J. H., Keum J. W. & Kim D. M. Expression screening of fusion partners from an E. coli genome for soluble expression of recombinant proteins in a cell-free protein synthesis system. PLoS One 6, e26875 (2011). 22073212 10.1371/journal.pone.0026875 PMC3206877

[b44] Kramer R. M., Shende V. R., Motl N., Pace C. N. & Scholtz J. M. Toward a molecular understanding of protein solubility: increased negative surface charge correlates with increased solubility. Biophys J 102, 1907–1915 (2012). 22768947 10.1016/j.bpj.2012.01.060 PMC3328702

[b45] Jones S., van Heyningen P., Berman H. M. & Thornton J. M. Protein-DNA interactions: A structural analysis. J Mol Biol 287, 877–896 (1999). 10222198 10.1006/jmbi.1999.2659

[b46] Jones S., Daley D. T., Luscombe N. M., Berman H. M. & Thornton J. M. Protein-RNA interactions: a structural analysis. Nucleic Acids Res 29, 943–954 (2001). 11160927 10.1093/nar/29.4.943 PMC29619

[b47] Crowley P. B. & Golovin A. Cation-pi interactions in protein-protein interfaces. Proteins 59, 231–239 (2005). 15726638 10.1002/prot.20417

[b48] Iyer G. H., Dasgupta S. & Bell J. A. Ionic strength and intermolecular contacts in protein crystals. Journal of Crystal Growth 217, 429–440 (2000).

[b49] Birtalan S. *et al.* The intrinsic contributions of tyrosine, serine, glycine and arginine to the affinity and specificity of antibodies. J Mol Biol 377, 1518–1528 (2008). 18336836 10.1016/j.jmb.2008.01.093

[b50] Arakawa T. *et al.* Suppression of protein interactions by arginine: a proposed mechanism of the arginine effects. Biophys Chem 127, 1–8 (2007). 17257734 10.1016/j.bpc.2006.12.007

[b51] Golovanov A. P., Hautbergue G. M., Wilson S. A. & Lian L. Y. A simple method for improving protein solubility and long-term stability. J Am Chem Soc 126, 8933–8939 (2004). 15264823 10.1021/ja049297h

[b52] Shukla D. & Trout B. L. Understanding the synergistic effect of arginine and glutamic acid mixtures on protein solubility. J Phys Chem B 115, 11831–11839 (2011). 21894928 10.1021/jp204462t

[b53] Koo T. Y. & Park T. H. Expression of recombinant human growth hormone in a soluble form in Escherichia coli by slowing down the protein synthesis rate. J Microbiol Biotechnol 17, 579–585 (2007). 18051267

[b54] Lawrence M. S., Phillips K. J. & Liu D. R. Supercharging proteins can impart unusual resilience. J Am Chem Soc 129, 10110–10112 (2007). 17665911 10.1021/ja071641y PMC2820565

[b55] Bairoch A. *et al.* The Universal Protein Resource (UniProt). Nucleic Acids Res 33, D154–159 (2005). 15608167 10.1093/nar/gki070 PMC540024

[b56] Wang G. & Dunbrack Jr R. L. PISCES: recent improvements to a PDB sequence culling server. Nucleic Acids Res 33, W94–98 (2005). 15980589 10.1093/nar/gki402 PMC1160163

[b57] Ahmad S. & Sarai A. Moment-based prediction of DNA-binding proteins. J Mol Biol 341, 65–71 (2004). 15312763 10.1016/j.jmb.2004.05.058

[b58] Nimrod G., Schushan M., Szilagyi A., Leslie C. & Ben-Tal N. iDBPs: a web server for the identification of DNA binding proteins. Bioinformatics 26, 692–693 (2010). 20089514 10.1093/bioinformatics/btq019 PMC2828122

[b59] Lo Conte L., Chothia C. & Janin J. The atomic structure of protein-protein recognition sites. J Mol Biol 285, 2177–2198 (1999). 9925793 10.1006/jmbi.1998.2439

[b60] Warwicker J. Simplified methods for pKa and acid pH-dependent stability estimation in proteins: removing dielectric and counterion boundaries. Protein Sci 8, 418–425 (1999). 10048335 10.1110/ps.8.2.418 PMC2144253

[b61] Neidhardt F. C. & Umbarger H. in Escherichia Coli and Salmonella: Cellular and Molecular Biology. 2nd edition, Vol. 1, (ed. Neidhardt, F. C.) (American Society of Microbiology, 1996).

